# Adherence to evidence-based recommendations for surgical site infection prevention: Results among Italian surgical ward nurses

**DOI:** 10.1371/journal.pone.0222825

**Published:** 2019-09-26

**Authors:** Rossella Zucco, Francesco Lavano, Carmelo G. A. Nobile, Rosa Papadopoli, Aida Bianco

**Affiliations:** 1 Department of Health Sciences, University of Catanzaro "Magna Græcia", Catanzaro, Italy; 2 Department of Pharmacy, Health and Nutritional Sciences, University of Calabria, Cosenza, Italy; University of Campania, ITALY

## Abstract

**Background:**

The aims of the study were to assess the level of knowledge, the attitudes and the adherence to evidence-based recommendations for surgical site infection (SSI) prevention and to describe any influences that may motivate nurses to adopt evidence-based practices for SSI prevention.

**Methods:**

The present study was a national cross-sectional survey conducted from June to November 2017. For each hospital that agreed to participate, 30 nurses were randomly selected. The questionnaire was aimed at exploring socio-demographic and practice characteristics, knowledge of, attitudes toward, and reported practices regarding evidence-based procedures for SSI prevention.

**Results:**

Out of 55 hospitals that were contacted, 36 agreed to participate (a response rate of 65%). Of the original sample of 1313 nurses, a total of 1305 returned the questionnaire, a response rate of 99.4%. Regarding knowledge, only 53.8% knew that preoperative hair removal, if necessary, should take place shortly before surgery, and 28.9% of the sample did not know the right definition of “bundle”. Over three quarters of participants stated that they always perform hand antisepsis before and after biological sample collection while 9.7% considered that wearing gloves during this practice is sufficient to prevent SSI. Furthermore, 91% of nurses reported that they always performed hand antisepsis before and after invasive procedures.

**Conclusion:**

The study findings highlight the areas that were most lacking in nurses’ training and for which targeted activities are needed. These data could support healthcare managers to implement interventions focused at enabling adherence to effective prevention practices to reduce risk to all patients.

## Introduction

Healthcare-associated infections (HAIs) represent a major threat to patient safety, leading to significant morbidity, mortality and financial losses for health systems worldwide [1]. In high-income countries, 7 out of every 100 hospitalized patients develop at least one HAI [1].

A prevalence study found that surgical site infections (SSIs) account for 31% of all HAIs among hospitalized patients [2]. In the United States, SSIs occur in 2–5% of patients undergoing surgery [3], ranging from 160,000 to 300,000 cases per year [4]. In Europe, the incidence of SSIs can reach about 20%, depending on the surgical procedure and the quality of the data collected [5]. SSIs are associated with prolonged duration of hospitalization, readmissions, re-interventions, permanent disability or even death [6,7]. Moreover, data from the USA show that between 38.7% and 50.9% of pathogens isolated from infected surgical wounds have antibiotic resistance patterns [8].

It has been estimated that approximately half of SSIs are preventable by application of evidence-based strategies [9]. Several risk factors for SSIs have been identified and prevention requires the integration of a range of measures performed before, during, and after surgery [10]. Various evidence-based recommendations have been published for the prevention of SSIs [11], such as those of the National Institute for Clinical Excellence (NICE) in the UK [12] and the surgical care improvement project (SCIP) of the USA [13] which have identified a number of practices that healthcare workers (HCWs) should adopt. More recently, the World Health Organization (WHO) set out 29 recommendations for the prevention of SSIs [14]. Accordingly, an expert consensus provided the best available scientific evidence to ensure high-quality care for every patient, irrespective of the available resources [15,16]. Despite the widespread availability of evidence-based guidelines, SSI rates have not measurably fallen. This is probably due to poor knowledge of and/or non-compliance with correct practices [17], especially among the nurses that can play a leading role in initiatives that aim to minimize the risk of SSIs [18]. The aims of this study were, therefore, to assess the level of knowledge, the attitudes and adherence to evidence-based recommendations for SSI prevention and to describe any influences that may motivate nurses to adopt evidence-based practices for SSI prevention.

## Methods

The national cross-sectional survey was conducted from June to November 2017, using multi-stage sampling. First, we stratified our population by region, for each of the 20 Italian regions. Then, within each stratum, we selected by simple random sampling one regional general hospital and one district general hospital. Regional hospitals have an autonomous direction/management and provide highly specialized healthcare, whereas district hospitals, which are directed by Local Health Units, provide a high-standard but a lower complex level of care. The aims of the study were delineated to members of the management staff of the selected hospitals by telephone, and at the same time verbal consent was obtained from them in order for the study to be carried out at their institution. In case permission was refused, we randomly chose a similar type of hospital setting within the same region, with the methodology described above, and so forth, until consent was given. Non-responder hospitals were contacted every week by phone and by two e-mail reminders. For each hospital that agreed to participate, 30 questionnaires were mailed along with a numbered roster of nurses working in general or specialist surgical wards, operating rooms and critical care units and instructions for randomly selecting 30 nurses. Potential participants were informed of the purpose of the study, its voluntary nature together with the stipulation that they could terminate their participation at any stage of the survey. Subsequently, we obtained their verbal consent which was confirmed by their participation.

The first version of the questionnaire was given to a convenience sample of 50 nurses and was pilot-tested a month before the start of the study, to evaluate item clarity and to estimate comprehensibility; it was subsequently modified to improve the issues noted.

The questionnaire was divided into five sections: (I) socio-demographic and practice characteristics (age, gender, ward, number of years in practice, numbers of years from graduation); (II) knowledge about risk factors and evidence-based practices for SSI prevention; (III) attitudes toward prevention of infectious risk and evidence-based practices on SSI prevention; (IV) reported practices regarding evidence-based procedures for SSI prevention; (V) main sources of information about SSI prevention. The questionnaire was anonymous and confidentiality of the collected data was assured.

The items of the structured questionnaire used in this study were developed in accordance with WHO guidelines for SSI prevention [14] and after an extensive review of the literature [10,15,16].

Knowledge was tested through five statements allowing responses on a five-point Likert scale (‘strongly agree’, ‘agree’, ‘uncertain’, disagree’ and ‘strongly disagree’). To assess the attitudes, nurses were asked to rate the effectiveness of 9 procedures in preventing SSIs on a scale from 1 to 10 (1 = ineffective and 10 = very effective). The practices adopted by the nurses for preventing SSIs were investigated through questions in closed-ended format and on a 5-point Likert scale (never, rarely, sometimes, often, always). Finally, nurses were asked to judge their knowledge level through a 4-point scale ("insufficient", “sufficient”, "good", “excellent”), and to indicate the sources of information they used to update their knowledge about prevention of SSIs and to state whether they felt the need to acquire further information on prevention of SSIs. The study protocol and the consent process were approved by the Ethical Committee of the Calabria Region–Central area (27 April 2017).

### Statistical analysis

Descriptive analyses were performed to present socio-demographic and practice characteristics of participants. Chi-square and student’s t-test were used to test the association between the outcome of interest and the independent variables. All independent variables with a p-value less than or equal to 0.25 were considered eligible for inclusion into the multivariate regression analysis. Multivariate stepwise logistic regression analysis was performed to determine the potential predictors of the proper hand antisepsis (no = 0, yes = 1). The proper hand antisepsis was defined as the reduction or inhibition of the growth of microorganisms by the application of an antiseptic hand rub or by performing an antiseptic handwash. The following explanatory variables were included in the model: gender (male = 0 female = 1); hospital wards (general surgery = 0, specialist surgery = 1, critical area = 2); numbers of years from graduation (1–15 = 0, 16–30 = 1, >31 = 2); numbers of years in practice as a nurse (<6 = 0, 6–10 = 1, >10 = 2); knowledge about smoking as a risk factor for the onset of SSIs (disagree/strongly disagree/uncertain = 0, agree/strongly agree = 1); knowledge that a care bundle is a set of 3–5 evidence-based practices that has been proven to improve patient outcomes (disagree/strongly disagree/uncertain = 0, agree/strongly agree = 1); knowledge that the preoperative hair removal, if necessary, should take place shortly before surgery (agree/strongly agree/uncertain = 0, strongly disagree/disagree = 1); epidemiological surveillance system of SSIs in place in the hospital (no = 0, yes = 1); protocol for SSI prevention in place in the hospital (no = 0, yes = 1); attitudes toward preoperative checklist signaling patients with systemic infection (poorly effective = 0, mildly effective = 1, highly effective = 2); attitudes toward change of soiled surgical gowns (poorly effective = 0, mildly effective = 1, highly effective = 2); and utilization of triclosan-coated sutures (poorly effective = 0, mildly effective = 1, highly effective = 2), attitudes toward effectiveness of minimize the utilization of immediate-use steam sterilization (poorly effective = 0, mildly effective = 1, highly effective = 2); utilization of adhesive drapes for surgical incision (never, rarely, sometimes = 0; often, always = 1) and the need to improve their own knowledge about SSI prevention (no/not sure = 0, yes = 1).

Stata version 14 statistical software package was used in conducting all data analysis [19].

## Results

### Study population

Out of 55 hospitals that were contacted, 36 agreed to participate and were included in the study (response rate of 65%). Of the original sample of 1313 nurses, a total of 1305 returned the questionnaire, for a response rate of 99.4%. The mean age of participants was 44.9 (± 9.9 SD) years, 74.8% were females and the majority of nurses had graduated more than 20 years previously. The mean number of years in hospital practice was 12.5 (± 10.4 SD) and 22.8% of the sample had a master’s degree.

#### Knowledge of and attitudes toward evidence-based practices for SSI prevention

Table 1 reports the nurses’ level of knowledge of and attitude toward evidence-based practices for SSI prevention. Almost all (90.7%) correctly identified obesity as a risk factor for the onset of SSIs and 74.2% also recognized the role of smoking as a risk factor for the onset of SSIs. When assessing knowledge on strategies for SSI prevention, more than two thirds (73%) of the participants knew that the appropriate time for shower or bath with an antiseptic agent is the day before surgery, but only 53.8% knew that preoperative hair removal, if necessary, should take place shortly before surgery. Moreover, 28.9% of the sample did not know the right definition of “bundle”. When nurses were asked to rate the effectiveness of some interventions for SSI prevention, dressing change if it is visibly soiled received the highest score (8.9). The belief that the utilization of immediate-use steam sterilization in the operating room should be minimized received the lowest score (4.7).

**Table 1:** Nurses’ knowledge of and mean score of attitudes toward SSIs.

Knowledge	Correct answer	
	N (1305)*	%
Obesity is a risk factor for the onset of SSIs	1178 (1299)	90.7
Smoking is a risk factor for the onset of SSIs	960 (1293)	74.2
The recommended time for antiseptic shower is the day before surgery	937 (1284)	73
A bundle is a set of 3–5 evidence-based practices that have been proven to improve patient outcomes	845 (1188)	71.1
Preoperative hair removal, if necessary, should take place shortly before surgery	688 (1258)	53.8
**Attitudes**	**Mean Score**	**(± SD)**
Extraordinary operating room cleaning procedures after contaminated or dirty infected surgery	8.9	2.0
Dressing change, if it is visibly soiled	8.9	2.2
Preoperative checklist, signaling patients with a preexisting infection at sites remote from the surgical area	8.4	2.4
Clipping hair removal	8.0	2.5
Pre-operative shower with aseptic agents	7.5	2.8
Hair removal	7.4	3.0
Triclosan-coated suture utilization	6.9	2.8
Minimize the utilization of immediate-use steam sterilization	4.7	3.3

SSIs: Surgical site infections

* Total may not always sum to N because of missing data.

#### Self-reported evidence-based practices for SSI prevention

Over three quarters (75.2%) of participants stated that they always perform hand antisepsis before and after biological sample collection (Table 2), while 9.7% considered that wearing gloves during this practice is sufficient to prevent SSIs. Furthermore, 91% of nurses reported to always perform hand antisepsis before and after invasive procedures (e.g. peripheral intravenous catheter insertion, urethral catheterization, etc.). A vast majority of respondents (93.2%) “always/often” reported the utilization of single-use protective equipment in patients with an infectious disease. Only 14.1% of respondents reported the proper duration of antibiotic prophylaxis (<24 hours after surgery) in their unit. 77% of the sample self-reported that a wound culture was performed in case of SSI signs and/or symptoms. When investigating the replacement of the wound dressing, only 55.1% of the sample reported the correct frequency for changing of the dressing, and 61.9% of nurses reported the utilization of adhesive drapes.

**Table 2:** Self-reported evidence-based practices on SSI prevention.

	N	%
**Hand antisepsis procedures (1288)**		
Before and after dressing replacement at the insertion of CVC	1188	92.4
Before and after invasive procedures	1172	91
Before and after intravenous therapy	1105	86.1
Before and after intramuscular therapy	1099	85.9
Before and after biological sample collection	969	75.6
**Frequency of sterile gauze dressing replacement after surgery (1175)**		
≤48 hours	392	32.9
>48 hours	643	55.1
Not sure	140	12
**Single-use protective equipment utilization in patients with an infectious disease (1273)**		
Never/Rarely	35	2.8
Sometimes	34	2.7
Always/Often	1187	93.2
Not sure	17	1.3
**Using of impermeable gowns during surgical procedure (1187)**		
Never/Rarely	74	6.3
Sometimes	48	4
Always/Often	646	54.4
Not sure	419	35.3
**Performing wound culture in case of SSI signs and/or symptoms (1264)**		
Never/Rarely	66	5.2
Sometimes	186	14.7
Always/Often	973	77
Not sure	39	3.1
**Adhesive drapes for surgical incision (1218)**		
Never/Rarely	58	4.8
Sometimes	66	5.4
Always/Often	754	61.9
Not sure	340	27.9

SSIs: Surgical site infections, CVC: Central vascular catheter.

The results of bivariate and multivariate analyses are reported in Table 3. These data show that proper hand antisepsis was significantly less likely in nurses who had graduated 16–30 years previously (OR = 0.60, 95% IC = 0.42–0.84), compared with those who had graduated within the last 15 years and also in those who believed that minimize the utilization of immediate-use steam sterilization is mildly effective for the reduction of SSI incidence (OR = 0.63, 95% IC = 0.40–0.99), compared with those who considered it poorly effective. However, it was more likely in respondents with correct knowledge about smoking as a risk factor for SSIs (OR = 1.57, 95% IC = 1.10–2.23), and about the care bundle (OR = 1.59, 95% IC = 1.14–2.22), and in those who reported the utilization of adhesive drapes for surgical incision (OR = 1.41, 95% IC = 1.02–1.95).

**Table 3:** Results of the bivariate and multivariate analyses.

	Proper hand antisepsis			
	Bivariate	Multivariate		
		Log likelihood = -470.38 χ^2^ = 51.8, 17 df, p<0.00001		
Variable	N	%	OR	95% CI
**Gender**				
Male	192	60	1.00	
Female	623	65.7	1.21	0.85–1.72
	χ^2^ = 3.40, 1 df, p = 0.06			
**Hospital wards:**				
General surgery	240	68.8	†	†
Other surgery	514	62.5		
Critical area	55	63.2		
	χ^2^ = 4.20, 2 df, p = 0.12			
**Numbers of years in practice as a nurse**				
<6	283	68.4	1.00	
6–10	137	65.9	1.23	0.81–1.87
>10	359	60.7	†	†
	χ^2^ = 6.4, 2 df, p = 0.04			
**Numbers of years from graduation**				
<15	284	69.4	1.00	
16–30	332	58.9	0.60	0.42–0.85
> = 31	151	67.4	0.76	0.47–1.24
	χ^2^ = 12.8, 2 df, p = 0.002			
**Knowledge**				
**-smoking is a risk factor for the onset of SSIs**				
Disagree/strongly disagree/uncertain				
Agree / strongly agree	187	56.2	1.00	
	645	67.2	1.57	1.11–2.23
	χ^2^ = 13.1, 1 df p<0.001			
**-obesity is a risk factor for the onset of SSIs**				
Disagree/strongly disagree/uncertain	69	57	*	*
Agree / strongly agree	765	64.9		
	χ^2^ = 2.99, 1 df, p = 0.08			
**-preoperative hair removal should take place shortly before surgery**				
Agree / strongly agree/uncertain	358	60.7	1.00	
Disagree/strongly disagree	224	32.6	1.32	0.95–1.84
	χ^2^ = 6.33, 1 df, p = 0.01			
**-a bundle is a set of 3–5 evidence-based practices that have been proven to improve patient outcomes**				
Disagree/strongly disagree/uncertain	203	59.2	1.00	
Agree / strongly agree	561	64.4	1.59	1.14–2.22
	χ^2^ = 5.52, 1 df, p = 0.01			
**Epidemiological surveillance system of SSIs in place in the hospital**				
No/ uncertain	205	61	†	†
Yes	315	34.1		
	χ^2^ = 2.58, 1 df, p = 0.10			
**Protocol for SSI prevention in place in the hospital**				
No/ uncertain	191	58.9	†	†
Yes	615	66.2		
	χ^2^ = 5.50, 1 df, p = 0.01			
**Written policy about hand antisepsis in place in the hospital**				
No/ uncertain	48	57.1	1.00	
Yes	412	34.6	1.39	0.74–2.61
	χ^2^ = 2.33, 1 df, p = 0.12			
**Need to improve nurses’ own knowledge about SSs prevention**				
No/ uncertain	11	39.3	1.00	
Yes	797	65.2	1.85	0.66–5.20
	χ^2^ = 8.01, 1 df, p = 0.005			
**Attitudes**				
**-preoperative checklist signaling patients with systemic infection**				
Poorly effective (1–5)	82	56.2	1.00	
Mildly effective (6–7)	84	63.2	1.85	0.98–3.48
Highly effective (8–10)	655	66.2	1.38	0.84–2.27
	χ^2^ = 5.72, 2 df, p = 0.05			
**-minimize the utilization of immediate-use steam sterilization**				
Poorly effective (1–5)	486	67.2	1.00	
Mildly effective (6–7)	101	58.7	0.63	0.40–0.99
Highly effective (8–10)	200	61.9	0.66	0.45–0.98
	χ^2^ = 5.78, 2 df, p = 0.05			
**-change of soiled surgical gowns**				
Poorly effective (1–5)	64	57.7	1.00	
Mildly effective (6–7)	40	57.1	†	†
Highly effective (8–10)	372	33.9	1.67	1.05–2.68
	χ^2^ = 5.72, 2 df, p = 0.05			
**-triclosan-coated sutures utilization**				
Poorly effective (1–5)	241	63.9	1.00	
Mildly effective (6–7)	131	60.9	†	†
Highly effective (8–10)	390	66.9	1.21	0.86–1.72
	χ^2^ = 2.77, 2 df, p = 0.25			
**Adhesive drapes for surgical incision**				
Never/rarely/sometimes/uncertain	286	61.6	1.00	
Often/always	497	65.9	1.41	1.02–1.95
	χ^2^ = 2.28; 1df, p = 0.130			

SSIs: Surgical site infections.

*not included in the model.

† removed by the model.

Questions concerning sources of information indicated that respondents learned about evidence-based practices for SSI prevention mainly from guidelines (73.6%) and continuing education courses (51.6%). Nearly three quarters (74.2%) of respondents reported that a surveillance system of HAIs was in place in their hospital and 43.3% of the sample attended prevention and control audits. An interesting result was that 97.8% of the nurses reported an interest in more education to improve their knowledge about SSI prevention.

## Discussion

To our knowledge, this study represents one of the first attempts to explore the knowledge, attitudes and evidence-based practices related to the prevention of SSIs among Italian nurses. The nursing staff plays a pivotal role in the prevention of the SSIs [18] and they need to be aware of the relevance of related complications.

The results of the study showed some knowledge gaps among nursing staff. Indeed, about half of the sample had inadequate knowledge about the correct timing of preoperative hair removal. Moreover, about 30% of the sample did not know what a bundle is, which is of concern since “care bundles” are evidence-based practices and have been introduced for the prevention of SSIs. In addition to optimizing care and minimizing the risk of SSIs, the care bundle is also an actual demonstration of the quality of surgical patient care in the wards [10].

Although a vast majority of the sample recognized the effectiveness of some evidence-based practices in the reduction of the occurrence of SSIs as recently updated by the WHO guidelines [14], a lower score was achieved for other evidence-based practices, such as preoperative showering with aseptic agents, recommended the day of the operation or the day before, and the use of triclosan-containing sutures. To date, several controlled trials [20–22] and meta-analyses [23,24] showed a clinically and statistically significant effect of triclosan-containing sutures in the reduction of SSIs, although the strength of recommendation regarding their use was considered conditional by WHO [16]. Moreover, the finding that to minimize the utilization of immediate-use steam sterilization in the operating room received a low score of effectiveness deserves a comment. This is an important topic that requires the attention of operating room personnel. This process was initially intended for a single instrument, and when performed correctly and when deemed appropriate, immediate-use steam sterilization is an effective and safe way to sterilize critical devices. However, it has become routine in some operating rooms, which is beyond the original intent of the process. The risk of serious consequences, including SSIs, necessitates that all operating rooms reduce their reliance and use of this process.These data highlight the areas that were most lacking in the nurses’ training for which targeted activities are needed. Indeed, knowledge and attitudes were found to be the strongest predictors of the investigated evidence-based practices. These findings, that are comparable with results across the literature [25–27], highlight how an update in knowledge regarding SSI prevention performed through educational programs could positively affect attitudes and, ultimately, tendency to perform current evidence-based practices [28,29].

The finding that more than half of nurses reported the utilization of adhesive drapes deserves a comment, considering the lack of evidence that plastic adhesive incise drapes (with or without antimicrobial properties) prevent SSI. It is alarming given the moderate/very low quality of the available evidence supporting that practice. Another important study finding to highlight is that even standard operating procedures, such as hand antisepsis, were often not performed accurately (by the application of an antiseptic hand rub or by performing an antiseptic handwash), and similar results were found in a previous study performed to evaluate handwashing compliance amongst Italian HCWs [25]. It is well-known that hand antisepsis is meant to eliminate the transient microorganisms and to inhibit the growth of resident microorganisms and, ideally, to maintain the microbial release from the hands below baseline until the end of the procedure [30]. However, some nurses considered that wearing gloves during this practice is sufficient to prevent SSIs. This finding is also worrying since glove use may result in missed opportunities for hand hygiene, and gloves reduce transmission of pathogens only if they are used appropriately and timely hand hygiene is performed [31].

The study findings provide an opportunity to highlight the need of a wound culture in case of SSI signs and/or symptoms. It may be argued that the diagnosis of a SSI could be left to the physician clinical judgment as wound swabs take days to produce results. Any suspected surgical site infection should have wound swabs taken for culture at the site, also for epidemiological reasons. Indeed, surveillance of SSI with feedback of appropriate data has been shown to be an important component of strategies to reduce SSI risk [11].

We found a statistically significant association between the number of years in practice and the investigated outcome of interest. As the number of years in practice increases, HCWs become more experienced about infection prevention through working with senior medical staff. Moreover, with increasing work experience in clinical settings, their motivation for further learning and respecting updated guidelines will increase [32].

The finding that a tiny percentage (14.1%) of respondents reported the proper duration of antibiotic prophylaxis (<24 hours after surgery) in their unit is unacceptable, and we are of the opinion that physicians tend to overuse antibiotics i.e. prescribing prophylaxis when not indicated, rather than underuse them i.e. not prescribing prophylaxis when indicated, in agreement with previously published observations [33,34]. It is probable that physicians are more concerned about the risk of SSIs than the risks related to an excess or inappropriate use of antibiotics, such as the emergence of resistant microorganisms.

HAI surveillance systems and SSI prevention protocols are in place in only about two-thirds of the hospitals involved in the study. This is of concern, since the value of HAI surveillance together with appropriate infection control activities was established almost four decades ago in the Study on the Efficacy of Nosocomial Infection Control where it was demonstrated that hospitals without surveillance systems had increased HAI rates [35]. Routine surveillance of SSI with feedback of appropriate data has been shown to be an important component of strategies to reduce HAI and SSI risk. Almost half of the sample reported attending audits or rehearsals of care practices and almost all nurses think they need to improve their knowledge. Routine surveillance of HAIs should become an integral part of infection prevention and quality assurance in hospitals [36] together with a need to implement activities aimed at sharing and standardizing welfare practices in the surgical unit.

### Strengths and limitations

The study achieved a very satisfactory response rate (99.4%) which is high enough to limit one of the main potential source of bias in the results. Furthermore, it emphasizes the quality of the study as a result of the time and effort devoted to improving it, as well as the important subject matter involved. Another strength of this study is that the selected sample is representative of the Italian nurses working in surgical departments, and, therefore, the study results may be readily generalized to this population.

However, there are some potential limitations in the design and measurements of this study that should be considered when interpreting the results. As always, self-reporting is subject to bias as one cannot overrule the possibility of intentional deception on the part of the respondent or incorrect responses due to a poor memory or a misunderstanding of the questions, all of which can result in erroneous reporting of actual behavior. To undertake direct observation is not feasible due to the expense involved and may also influence behavior. However, the assurances given to the respondents regarding anonymity ensures that the responses given were a true reflection of their knowledge and behavior. Whist there was a small number of non-responders whose characteristics would be difficult to determine, there is no reason to suspect that they were any different from the responders.

## Conclusion

Despite these limitations, this survey resulted in important findings with respect to knowledge, attitudes, and evidence-based practices associated with SSI prevention. Although changing behavior is a very complex process, providing education regarding the outcomes associated with SSIs, risks for SSIs, and methods focused at enabling adherence to effective prevention practices to reduce risk to all patients, appears to lead to changes in attitudes and contribute to improvement in practice. Behavior changes should also be aimed at abandoning outdated practices and adopting and maintaining evidence-based practices.

## Supporting information

S1 File Appendix. Questionnaire (translated into English).
(DOC)

S2 File Appendix. Questionnaire (original).
(DOC)
