# Supplementary material for: Adherence to evidence-based recommendations for surgical site infection prevention: Results among Italian surgical ward nurses
Source: PLoS One. 2019 Sep 26;14(9):e0222825. doi: 10.1371/journal.pone.0222825 (PMC6762080; doi:10.1371/journal.pone.0222825)
Supplement: S1 File — Questionnaire (translated into English). (DOC) [file pone.0222825.s001.doc]

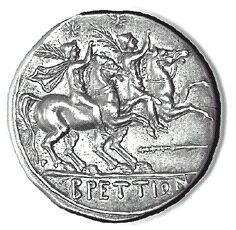


UNIVERSITA’ DEGLI STUDI DI CATANZARO

“MAGNA GRÆCIA”

HEALTH SCIENCE DEPARTMENT

SCHOOL OF MEDICINE

Graduate school in Hygiene and Preventive Medicine

**KNOWLEDGE, ATTITUDE AND REPORTED EVIDENCE-BASED PRACTICES ABOUT SSI PREVENTION AMONG ITALIAN HEALTHCARE WORKERS IN SURGERY WARDS**

**A. SOCIO-DEMOGRAPHIC AND PRACTICE CHARACTERISTICS**

**A1.** Birth year ___________ **A2.**Gender 🗆 M 🗆F **A3.** Graduation Year______________________

**A4.** Specify the hospital in which you currently work _________________________________________

**A5.** Specify the surgical ward in which you currently work _____________________________________

**A6** Did you get a master's degree? 🗆Yes 🗆NO

**A7.** Specify the numbers of years in practice as a nurse in your ward (_____________)

**B. KNOWLEDGE**

**Knowledge about risk factors and evidence-based practices for SSI prevention**

***choose only one answer***

|  | Strongly agree | Agree | Uncertain | Disagree | Strongly disagree |
| --- | --- | --- | --- | --- | --- |
| B1. Obesity is a risk factor for the onset of SSI | 🗆 | 🗆 | 🗆 | 🗆 | 🗆 |
| B2. Smoking is a risk factor for the onset of SSI | 🗆 | 🗆 | 🗆 | 🗆 | 🗆 |
| B3. Preoperative hair removal, if necessary, should take place shortly before surgery | 🗆 | 🗆 | 🗆 | 🗆 | 🗆 |
| B4. The recommended time for antiseptic shower is the day before surgery | 🗆 | 🗆 | 🗆 | 🗆 | 🗆 |
| B5. A bundle is a set of 3-5 evidence-based practices that, when performed collectively, have been proven to improve patient outcomes | 🗆 | 🗆 | 🗆 | 🗆 | 🗆 |

| **C. ATTITUDES TOWARD PREVENTION OF INFECTIOUS RISK AND EVIDENCE-BASED PRACTICES ON SSI PREVENTION**  ***choose only one answer***  **Using a scale from 1 to 10, how would you classify the effectiveness of following practices to reduce the incidence of SSI? (1= ineffective and 10=** **very effective)** | | | | | | | | | | |
| --- | --- | --- | --- | --- | --- | --- | --- | --- | --- | --- |
|  | 1 | 2 | 3 | 4 | 5 | 6 | 7 | 8 | 9 | 10 |
| C1. A pre-operative shower with antiseptic agents | 🗆 | 🗆 | 🗆 | 🗆 | 🗆 | 🗆 | 🗆 | 🗆 | 🗆 | 🗆 |
| C2. Hair removal | 🗆 | 🗆 | 🗆 | 🗆 | 🗆 | 🗆 | 🗆 | 🗆 | 🗆 | 🗆 |
| C3. Clipping hair removal | 🗆 | 🗆 | 🗆 | 🗆 | 🗆 | 🗆 | 🗆 | 🗆 | 🗆 | 🗆 |
| C4. Preoperative checklist, signaling patients with a preexisting infection at sites remote from the surgical area | 🗆 | 🗆 | 🗆 | 🗆 | 🗆 | 🗆 | 🗆 | 🗆 | 🗆 | 🗆 |
| C5. Minimize the utilization of immediate-use steam sterilization | 🗆 | 🗆 | 🗆 | 🗆 | 🗆 | 🗆 | 🗆 | 🗆 | 🗆 | 🗆 |
| C6. Dressing change if it is visibly soiled | 🗆 | 🗆 | 🗆 | 🗆 | 🗆 | 🗆 | 🗆 | 🗆 | 🗆 | 🗆 |
| C7. Triclosan-coated sutures utilization | 🗆 | 🗆 | 🗆 | 🗆 | 🗆 | 🗆 | 🗆 | 🗆 | 🗆 | 🗆 |
| C8. Extraordinary operating room cleaning procedures after contaminated or dirty infected surgery | 🗆 | 🗆 | 🗆 | 🗆 | 🗆 | 🗆 | 🗆 | 🗆 | 🗆 | 🗆 |

**D. REPORTED PRACTICES REGARDING EVIDENCE-BASED PROCEDURES FOR SSI PREVENTION**

***mark the answer that best reflects your behavior or the organization of your work place***

D1. How many times, on average, do you wash your hands during a work shift?

D2. When do you perform antiseptic hand washing how long does it take?

 1-10 seconds  10-30 seconds  30-40 seconds

 >=60 seconds 1-2 minutes  2-5 minutes

D3. In your ward, how many hours after the surgery is the antibiotic prophylaxis suspended?

** Specify (___) hours 🗆 I don't know, it's not within my competence

D4. In your ward after how many days the sterile dressings with gauze used to protect the incisions closed in the first intention are replaced?

1 day 2 days 3 days Other (specify____) I don't know, it's not within my competence

|  | Before | Before & After | After | The use of gloves is sufficient |
| --- | --- | --- | --- | --- |
| D5. Mark for each listed procedures, the moments when you perform handwashing |  |  |  |  |
| 1. Intramuscular therapy | 🗆 | 🗆 | 🗆 | 🗆 |
| 1. Biological samples collecting | 🗆 | 🗆 | 🗆 | 🗆 |
| 1. Intravenous therapy | 🗆 | 🗆 | 🗆 | 🗆 |
| 1. Invasive procedures | 🗆 | 🗆 | 🗆 | 🗆 |
| 1. Dressings replacement at the insertion of CVC | 🗆 | 🗆 | 🗆 | 🗆 |

|  | Never | Rarely | Sometimes | Often | Always | Not sure |
| --- | --- | --- | --- | --- | --- | --- |
| D6. Do you utilize single-use protective equipment in patients with an infectious disease? | 🗆 | 🗆 | 🗆 | 🗆 | 🗆 | 🗆 |
| D7. Do you use impermeable gowns during surgical procedure? | 🗆 | 🗆 | 🗆 | 🗆 | 🗆 | 🗆 |
| D8. Are adhesive drapes used for surgical incision? | 🗆 | 🗆 | 🗆 | 🗆 | 🗆 | 🗆 |
| D9. If the patient has signs and/or symptoms of SSI is the wound culture swab performed? | 🗆 | 🗆 | 🗆 | 🗆 | 🗆 | 🗆 |

**E. MAIN SOURCES OF INFORMATION ABOUT SSI PREVENTION**

|  | Yes | No | Not sure |
| --- | --- | --- | --- |
| E1. Is there an epidemiological surveillance system of SSI in place in the hospital? | 🗆 | 🗆 | 🗆 |
| E2. Is there a protocol for SSI prevention in place in the hospital? | 🗆 | 🗆 | 🗆 |
| E3. A common operating procedure / instruction was identified for all healthcare workers for the following practices: | | | |
| Hand antisepsis | 🗆 | 🗆 | 🗆 |
| Patient preparation for surgery | 🗆 | 🗆 | 🗆 |
| Surgical dressing management | 🗆 | 🗆 | 🗆 |
| E4. Does your hospital organize audits or meetings to review evidence-based practices on SSI prevention? | 🗆  (go next) | 🗆 (go to E5) | 🗆  (go to E5) |

E4.1 How often? 🗆 Weekly 🗆 Monthly 🗆 Every trimester 🗆 Every semester

🗆 Yearly 🗆 Specify________________________________________

E4.2 What are the professional figures involved? 🗆 Only doctors 🗆 Doctors and nurses 🗆 Others

E5. From which sources do you acquire information about evidence-based practices on SSI prevention? 🗆 Nothing 🗆 Guidelines 🗆 Continuing education courses 🗆 Sc. Societies 🗆 Scientific magazines

E6. How would you rate your knowledge about proven practices on SSI prevention?

🗆 Insufficient 🗆 Sufficient 🗆 Good 🗆 Excellent

E7. Do you think it is important to improve your current knowledge? 🗆Yes 🗆No 🗆I don’t know

**The questionnaire is finished, however if you think there is something to add use the space below.**

__________________________________________________________________________________________________________________________________________________________

__________________________________________________________________________________________________________________________________________________________

**THANKS FOR COLLABORATION**
