# Supplementary material for: Adherence to evidence-based recommendations for surgical site infection prevention: Results among Italian surgical ward nurses
Source: PLoS One. 2019 Sep 26;14(9):e0222825. doi: 10.1371/journal.pone.0222825 (PMC6762080; doi:10.1371/journal.pone.0222825)
Supplement: S2 File — Questionnaire (original). (DOC) [file pone.0222825.s002.doc]

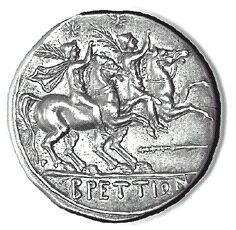


UNIVERSITA’ DEGLI STUDI DI CATANZARO

“MAGNA GRÆCIA”

**DIPARTIMENTO DI SCIENZE DELLA SALUTE**

**SCUOLA DI MEDICINA E CHIRURGIA**

Scuola di specializzazione in Igiene e Medicina Preventiva

**CONOSCENZE, ATTITUDINI E COMPORTAMENTI DEGLI OPERATORI SANITARI SULLE PRATICHE DI PROVATA EFFICACIA PER LA PREVENZIONE DELLE INFEZIONI DEL SITO CHIRURGICO**

**A. INFORMAZIONI ANAGRAFICHE E PROFESSIONALI**

**A1.** Anno di nascita ___________ **A2.**Sesso 🗆 M 🗆F **A3.**Anno di Laurea/Diploma_______

**A4.** Presidio Ospedaliero di appartenenza ____________________________________________

**A5.** Unità Operativa di appartenenza ________________________________________________

**A6** Ha conseguito un master? 🗆SI 🗆NO

**A7.** Da quanti anni svolge attività presso questa U.O. (specifichi_________________________)

**B. CONOSCENZE**

**conoscenza delle pratiche di provata efficacia per la prevenzione delle ISC**

*Scelga una sola risposta per ciascuna affermazione*

|  | Fortemente d’accordo | D’accordo | Incerto | In disaccordo | Fortemente in disaccordo |
| --- | --- | --- | --- | --- | --- |
| B1. L’obesità rappresenta un fattore di rischio per l’insorgenza di ISC | 🗆 | 🗆 | 🗆 | 🗆 | 🗆 |
| B2. Il fumo rappresenta un fattore di rischio per l’insorgenza di ISC | 🗆 | 🗆 | 🗆 | 🗆 | 🗆 |
| B3. La tricotomia va eseguita immediatamente prima dell’intervento chirurgico | 🗆 | 🗆 | 🗆 | 🗆 | 🗆 |
| B4. La doccia con antisettico va eseguita il giorno prima dell’intervento chirurgico | 🗆 | 🗆 | 🗆 | 🗆 | 🗆 |
| B5. Il bundle è un insieme di pratiche di provata efficacia, generalmente da 3 a 5, che se effettuate insieme migliorano la qualità e l’esito degli interventi sanitari | 🗆 | 🗆 | 🗆 | 🗆 | 🗆 |

| **C. ATTITUDINI**  **attitudini sulla prevenzione e controllo del rischio infettivo**  **In una scala da 1 a 10 come classificherebbe l’efficacia delle seguenti pratiche per la riduzione dell’incidenza delle ISC? (1 indica INEFFICACE e 10 indica MOLTO EFFICACE)**  ***Scelga una sola risposta per ciascuna affermazione*** | | | | | | | | | | |
| --- | --- | --- | --- | --- | --- | --- | --- | --- | --- | --- |
|  | 1 | 2 | 3 | 4 | 5 | 6 | 7 | 8 | 9 | 10 |
| C1. Una doccia preoperatoria con antisettico prima dell’intervento | 🗆 | 🗆 | 🗆 | 🗆 | 🗆 | 🗆 | 🗆 | 🗆 | 🗆 | 🗆 |
| C2. La tricotomia del paziente | 🗆 | 🗆 | 🗆 | 🗆 | 🗆 | 🗆 | 🗆 | 🗆 | 🗆 | 🗆 |
| C3. Tricotomia mediante clipper | 🗆 | 🗆 | 🗆 | 🗆 | 🗆 | 🗆 | 🗆 | 🗆 | 🗆 | 🗆 |
| C4. Check-list preoperatoria per segnalare pazienti con eventuale infezione in altra sede | 🗆 | 🗆 | 🗆 | 🗆 | 🗆 | 🗆 | 🗆 | 🗆 | 🗆 | 🗆 |
| C5. Limitare l’utilizzo della sterilizzazione “flash” | 🗆 | 🗆 | 🗆 | 🗆 | 🗆 | 🗆 | 🗆 | 🗆 | 🗆 | 🗆 |
| C6. Cambio delle medicazioni se macroscopicamente sporche | 🗆 | 🗆 | 🗆 | 🗆 | 🗆 | 🗆 | 🗆 | 🗆 | 🗆 | 🗆 |
| C7. Utilizzo di fili da sutura rivestiti di Triclosan | 🗆 | 🗆 | 🗆 | 🗆 | 🗆 | 🗆 | 🗆 | 🗆 | 🗆 | 🗆 |
| C8. Procedure straordinarie di lavaggio della sala operatoria dopo interventi chirurgici contaminati o sporchi | 🗆 | 🗆 | 🗆 | 🗆 | 🗆 | 🗆 | 🗆 | 🗆 | 🗆 | 🗆 |

**D. COMPORTAMENTI**

**pratiche di prevenzione delle ISC che si svolgono quotidianamente nella attività chirurgica**

*Indichi la risposta che meglio rispecchia il suo comportamento o l’organizzazione all’interno della sua U.O.*

D1. Quante volte, in media, durante un turno lavorativo si lava le mani? ___________________

D2. Quando effettua il lavaggio sociale delle mani saprebbe indicare, orientativamente, quanto dura?

 Meno di 10 secondi  Tra 10 e 30 secondi  Tra 30 e 40 secondi

 Almeno 60 secondi  Almeno 1-2 minuti  Tra i 2 e gli 5 minuti

D3. Nella sua U.O. dopo quante ore dall’intervento viene sospesa la profilassi antibiotica?

** Specifichi n. ore(___) 🗆 Non so, non rientra tra le mie competenze

D4. Nella sua U.O., dopo quanti giorni vengono sostituite le medicazioni sterili utilizzate per proteggere le incisioni chiuse in prima intenzione?

1 giorno 2 giorni 3 giorni Altro (specifichi____) Non so, non rientra fra le mie competenze

|  | Before | Before & After | After | The use of gloves is sufficient |
| --- | --- | --- | --- | --- |
| D5. Indichi, per ciascuna delle procedure elencate, quando ricorre al lavaggio delle mani |  |  |  |  |
| 1. Somministrazione di terapie intramuscolo | 🗆 | 🗆 | 🗆 | 🗆 |
| 1. Prelievo di materiale biologico | 🗆 | 🗆 | 🗆 | 🗆 |
| 1. Somministrazione di terapie endovenose | 🗆 | 🗆 | 🗆 | 🗆 |
| Procedure invasive | 🗆 | 🗆 | 🗆 | 🗆 |
| 1. Gestione delle medicazioni all’emergenza dei dispositive invasivi | 🗆 | 🗆 | 🗆 | 🗆 |

|  | Mai | Raramente | Qualche volta | Spesso | Sempre | Non so |
| --- | --- | --- | --- | --- | --- | --- |
| D6. Utilizza dispositivi di protezione individuale monouso sui pazienti infetti | 🗆 | 🗆 | 🗆 | 🗆 | 🗆 | 🗆 |
| D7. Indossa divise impermeabili durante le procedure chirurgiche? | 🗆 | 🗆 | 🗆 | 🗆 | 🗆 | 🗆 |
| D8. Per l’incisione chirurgica vengono utilizzati teli adesivi? | 🗆 | 🗆 | 🗆 | 🗆 | 🗆 | 🗆 |
| D9. In presenza di segni e/o sintomi di ISC vengono eseguiti tamponi colturali della ferita? | 🗆 | 🗆 | 🗆 | 🗆 | 🗆 | 🗆 |

**E. FONTI INFORMATIVE SULLA PREVENZIONE DELLE ISC**

|  | Si | No | Non so |
| --- | --- | --- | --- |
| E1. Presso la sua azienda/presidio ospedaliero è attivo un sistema di sorveglianza epidemiologica delle infezioni correlate all’assistenza?? | 🗆 | 🗆 | 🗆 |
| E2. E’ presente nella sua azienda/presidio ospedaliero un protocollo incentrato sulla prevenzione delle ISC? | 🗆 | 🗆 | 🗆 |
| E3. E’ stata identificata una procedura/istruzione operativa comune a tutti gli operatori per le seguenti pratiche: | | | |
| Lavaggio antisettico delle mani | 🗆 | 🗆 | 🗆 |
| Preparazione del paziente all’intervento chirurgico | 🗆 | 🗆 | 🗆 |
| Gestione delle medicazioni chirurgiche | 🗆 | 🗆 | 🗆 |
| E4. Vengono organizzati nella sua U.O. audit o incontri di revisione delle pratiche assistenziali sulla prevenzione e controllo delle ISC? | 🗆  (segue E4.1) | 🗆 (segue E5) | 🗆  (segue E5) |

E4.1 Con quale frequenza? 🗆 Settimanale 🗆 Mensile 🗆 Trimestrale 🗆 Semestrale 🗆 Annuale 🗆 Specifichi________________________________

E4.2 Quali figure professionali sono coinvolte? 🗆 Solo medici 🗆 Medici e infermieri 🗆 Altri (specifichi_____________________)

E5. Da quali fonti acquisisce informazioni sulle pratiche evidence-based per la prevenzione delle ISC? 🗆 Nessuna 🗆 Linee-Guida 🗆 Corsi di formazione 🗆 Società scientifiche 🗆 Riviste scientifiche

E6. Come giudica la sua conoscenza sulle pratiche di provata efficacia nella prevenzione delle ISC? 🗆 Insufficiente 🗆 Sufficiente 🗆 Buona 🗆 Eccellente

E7. Ritiene importante migliorare le sue attuali conoscenze? 🗆Si 🗆No 🗆Non so

**Il questionario è finito, tuttavia se ritiene che ci sia qualcosa da aggiungere utilizzi lo spazio sottostante.**

__________________________________________________________________________________________________________________________________________________________

**GRAZIE PER LA COLLABORAZIONE**
